# Modeling the Energy Performance of Event-Driven Wireless Sensor Network by Using Static Sink and Mobile Sink

**DOI:** 10.3390/s101210876

**Published:** 2010-12-02

**Authors:** Jiehui Chen, Mariam B. Salim, Mitsuji Matsumoto

**Affiliations:** 1 Global COE Program International Research and Education Center for Ambient SoC, Waseda University, Tokyo, 169-8555, Japan; 2 Graduate School of Global Information and Telecommunication Studies, Waseda University, Tokyo, 169-0051, Japan; E-Mails: msalim@fuji.waseda.jp (M.B.S.); mmatsumoto@waseda.jp (M.M.)

**Keywords:** wireless sensor network, modeling the energy performance, event-driven, mobile sink

## Abstract

Wireless Sensor Networks (WSNs) designed for mission-critical applications suffer from limited sensing capacities, particularly fast energy depletion. Regarding this, mobile sinks can be used to balance the energy consumption in WSNs, but the frequent location updates of the mobile sinks can lead to data collisions and rapid energy consumption for some specific sensors. This paper explores an optimal barrier coverage based sensor deployment for event driven WSNs where a dual-sink model was designed to evaluate the energy performance of not only static sensors, but Static Sink (SS) and Mobile Sinks (MSs) simultaneously, based on parameters such as sensor transmission range *r* and the velocity of the mobile sink *v*, *etc.* Moreover, a MS mobility model was developed to enable SS and MSs to effectively collaborate, while achieving spatiotemporal energy performance efficiency by using the knowledge of the cumulative density function (cdf), Poisson process and M/G/1 queue. The simulation results verified that the improved energy performance of the whole network was demonstrated clearly and our eDSA algorithm is more efficient than the static-sink model, reducing energy consumption approximately in half. Moreover, we demonstrate that our results are robust to realistic sensing models and also validate the correctness of our results through extensive simulations.

## 1. Introduction

Wireless Sensor Networks (WSNs) equipped with extremely small, low cost sensors that possess sensing, signal processing and wireless communication capacities are highly capable of performing monitoring applications. In conventional WSNs, a dense static sensor deployment is implicitly required. Subsequently, there arises a fundamental problem in WSNs with static topology: the non-uniformity of energy consumption among the sensors. In fact, the nearer a sensor lies in relation to the sink, the faster its energy will be depleted. In case of sensor failure or malfunction around a sink, the network connectivity and coverage may not be guaranteed. Intuitively, there are two solutions to the above problems. On the one hand, if some sensors withdraw from the network due to energy exhaustion such that the network loses the necessary connectivity and sensing coverage, other supplementary sensors must be deployed. On the other hand, the sensors should be capable of finding and reaching the sink in possibly different positions, whether there are multiple sinks or the sink is able to change its location. The first approach is frequently related to the design of mobile robotics, therefore we will focus our efforts on the second one: the use of multiple mobile sinks. It is envisaged that in the near future, very large scale networks consisting of both mobile and static nodes will be deployed for mission-critical applications ranging from environmental monitoring to emergency search-and-rescue operations. Energy is identified as the most crucial resource in sensor networks due to the difficulty of recharging batteries of thousands of devices in remote or hostile environments. In this paper, we show that it is possible to achieve considerable savings in energy consumption expended on communication to mobile sinks at the expense of a moderate increase in message delivery delay. Exploration of this trade-off is the main principle that underlies the design of our algorithm.

It is known to all that the deployment should result in configurations that not only provide good “sensor coverage” but also satisfy certain global (e.g., network connectivity) constraints. In [1] sensor coverage problems were studied and categorized into three types: *area coverage*, *point coverage*, and *barrier coverage*. The objective of the first, area coverage, is to maximize the coverage for a region of interest. The objective of point coverage is similar, but it is to cover a set of points. The latter, barrier coverage, aims to minimize the probability of undetected penetration through a sensor network. The choice of using a particular coverage measurement depends on the purpose of a sensor network. For instance, if the purpose is to monitor moving objects in a field, barrier coverage is more suitable. To measure barrier coverage, [2] defined the worst- and best-case coverage. They proposed two centralized algorithms to solve these problems. The best-case coverage algorithm was later extended to a distributed localized one in [3]. Based on the optimal multi-hop network coverage solution, we investigated detecting an event where sensors may need to aggregate the data. Therefore, a Mobile Sink (MS) was introduced into the network clustered in a similar way as in our previous work [4]. Regarding modeling the energy performance, in the energy model for gathered data transmission, MS mobility and load balancing critical factors.

## 2. Related Work

The energy consumption model in [5] established that the energy consumed by transmitting a unit of data is the same for each node, whereas energy performance is still hard to evaluate based on elusory communication distance measurements. In [6] the problem of maintaining the distance measurement of the best- and worst-case coverage of a network was studied. For any given ɛ, ɛ > 0, the algorithm could maintain a (1 + ɛ) approximation on the best-case coverage distance and a ( $\sqrt{2}+ɛ$)approximation on the worst-case coverage distance of a sensor network. To find an optimal sensor deployment, the search space should contain not only the Delaunay triangulation of sensors but also the edges formed by any two sensors. Our solution finds the best locations for new sensors in polynomial time so that the placement of new sensors can optimally improve the best-case coverage of a sensor network. The thought behind the algorithm is based on computational geometry and graph theory, including Voronoi diagrams, Delaunay triangulation, and graph search algorithms for coverage calculation. Regarding the use of MS, [8] proposed a dual-sink protocol which shows that when it scales up, the network using Dual-Sink enjoys steady lifetime improvement and energy saving from sink mobility, whereas the network with only one MS performs no better than the network with a single SS. [7] designed a movement circle trajectory for MS, all the sensed data are forwarded into the annularity area and then be collected by the MS. The proposed MA (Movement in an Annulus) was proven to be efficient compared with RM (Random Movement), PM (Peripheral Movement) and SM (Static-based Model) [8]. In [9] a sink mobility supported route protocol for environment monitoring called Patrol Grid Protocol (PGP) was proposed, that was proven to be better than TWO-Tier Data Dissemination (TTDD) [10] in terms of less overhead and delay rate. PGP is more suitable for urgent events and query-driven mode. The authors in [11] proposed a novel cooperative forwarding process and presented a novel cooperative contention-based forwarding (CCBF) that extends the scope of cooperation and attains the full potential of cooperative forwarding at the expense of sending one additional control message on demand. CCBF employed a retransmission mechanism to significantly decrease the end-to-end hop counts and energy efficiency and latency as well as the packet loss ratio. In [12] the use of two mobile sinks in the event-driven WSN was proposed. It prolongs the network lifetime and reduces the data delay in a different mobility patterns. In [13] an algorithm called EMOSEN that introduced a multi-radio enabled mobile into WSN to investigate the heterogeneity, sensor mobility and capacity gain was proposed. In [14] an efficient Query-based data collection scheme (QBDCS) that considers a moving mobile sink queries a specific area or a point of interest for data collection is proposed. Due to the mobility of the mobile sink, the Query and Response should be in different routes. QBDCS chooses the optimal timing to send the query packet and tailors the routing mechanism for partial sensor nodes forwarding packets with minimum energy consumption and delivery latency. In [15] the authors investigated the impacts of different features and behavior of mobile sinks on hybrid wireless sensor networks. Analysis and simulation results showed that, instead of deploying as many mobile sinks as possible, choosing appropriate number, transmission range, velocity and gathering mode of the sink nodes can significantly decrease the average end-to-end data delivery delay and improve the energy conservation. In [16] a cluster based data dissemination (CBDD) scheme was proposed to divide the communication between mobile sinks and source sensors into inter-cluster and intra-cluster communication phases and limit the disruption of the data dissemination path within a cluster which does not require any location algorithms. [17] developed a simple and efficient data delivery schemes tailored for DFT-MSN, which has several unique characteristics such as sensor mobility, loose connectivity, fault tolerability, delay tolerability, and buffer limit with an optimized flooding scheme that minimizes transmission overhead in flooding.

In [18] an optimum predefined mobility trajectory of the sink that was explored to balance the two performance metrics was given. The authors focused on a lattice-based analytical model to understand performance as system and mobility parameters are scaled. The authors of [19] proposed a new solution with adaptive location updates for mobile sinks to resolve rapid energy consumption and collision problems. The fantasy of this paper is that it only needs to broadcast its location information within a local area other than among the entire network. The authors of [20] investigated the benefits of a heterogeneous WSN architecture with rich mobile sensors and static sensors. Their algorithms show many significant effects on the computational network lifetime and performance. However, the shortcoming of this approach is that they assumed all the sensors in the network to be aware of the location of the mobile sensor. Our paper explores an optimal barrier coverage based sensor deployment for event driven WSNs where a dual-sink model was designed to evaluate the energy performance of not only static sensors, but Static Sinks (SS) and Mobile Sinks (MSs) simultaneously based on parameters such as sensor transmission range *r* and the velocity of the mobile sink *v etc.* Moreover, a MS mobility model was developed to enable SS and MSs to effectively collaborate, while achieving a set of spatiotemporal energy performance efficiency by using the knowledge of the cumulative density function (cdf), Poisson process and M/G/1 queue.

The remainder of this paper is organized as follows: the next section presents the proposed efficient Dual Sink Algorithm for an event driven Wireless Sensor Network (eDSA) with optimized barrier coverage design. Section 4 generally illustrates the network model and detailed solutions for modeling the energy performance and Section 5 shows the simulation results. Finally, Section 6 concludes the paper.

## 3. eDSA = Efficient Dual Sink Algorithm for Event-Driven WSNs by using SS and MS

### 3.1. Optimized Barrier Coverage Design

**Figure 1.**
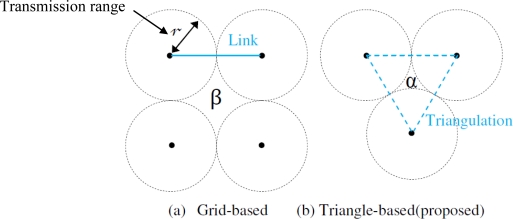
Detection capacity-based sensor deployment.

Although maintaining full sensing coverage guarantees an immediate response to intruding targets, sometimes it is not favorable due to its high energy consumption. We have investigated a new and more efficient approach for deploying sensor nodes in a large scale network area. To monitor an area, a WSN should achieve a certain level of detection performance. Due to the considerably high cost in a given monitoring area, better detection capacity and communication coverage is critical to sequential deployment of sensors. In this paper, a new sensor deployment was developed (see Figure 1) to improve barrier coverage.

**Theorem 1.** Let A denote the area and *f*(A) denote barrier coverage, namely the fraction of the area that is in the sensing area of one or more sensors where sensors can provide a valid sensing measurement and Γ is the cartographic representation of area. Then:

(1) $$\Gamma_{f(\beta )}\gg \Gamma_{f(\alpha )}\text{in}\;\text{G}=(\text{V},\;\text{E})\;\text{where}\;\text{E}\neq \emptyset$$

**Proof**: In the literature, the majority of researchers prefer grid-based sequential sensor deployment [see Figure 1(a)]. Instinctively, we see that Γ_f(__β__)_ is more efficient than Γ_f(__α__)_. The computations are as follows:

(2) $$\Gamma_{f(\beta )}={\left(2r\right)}^{2}-4(\frac{\pi {\text{r}}^{2}}{4})=(4-\pi )r^{2}\approx 0.86r^{2}$$

(3) $$\Gamma_{f(\alpha )}=(\sqrt{3}-\frac{\pi }{2})r^{2}\approx 0.1512r^{2}$$

Since the calculation work is easy, we have skipped the computation procedure and go directly to the result. The unit difference is obviously given by approximately 0.71*r*^2^. Although the difference is indistinctive when the value of *r* is small enough, for monitoring applications, accuracy is a vital consideration. The smaller the value of Γ_*f*(.)_ is, the higher possibility that a moving object will not be detected, therefore Figure 1(b) has better detection capacity than Figure 1(a).

**Theorem 2**. In a hierarchical network architecture, let H_v_ be a threshold hop distance and ${\text{p}}_{\text{v}}^{\text{up}}$, ${\text{p}}_{\text{v}}^{\text{same}}$ and ${\text{p}}_{\text{v}}^{\text{lower}}$ denotes the possible existence of CHs at the upper, same and lower layer respectively. The proposed Triangle-based is more suitable for our monitoring network in term of higher density of hop distance neighborhood.

**Figure 2.**
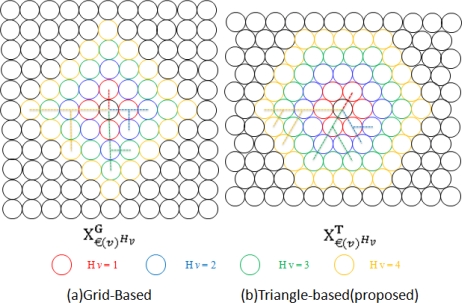
New sensor deployment based on higher density of hop distance neighborhood.

**Proof**: Figure 2 clearly shows that the Triangle-based approach has more relay one hop neighbors €(v) (self-defined) to relay to than a Grid-based one at a rate of 6:4. For multi-hop transmission, when receiving a message, a sensor (N_v_) should relay it to another sensor with a certain energy consumption cost. The sensor to relay should be one at the higher layer compared to N_v_. ${\text{H}}_{\text{v}}^{\text{up}}$, ${\text{H}}_{\text{v}}^{\text{same}}$ and ${\text{H}}_{\text{v}}^{\text{lower}}$ represent the number of hops on the shortest routing path from N_v_ to a sensor at the upper, same and lower layer, respectively. On the other hand, within a certain hop distance, the higher possibility of existing sensors to relay, the better. Therefore, the focus is to find out which one has more €(v)^H_v_^ between Figure 2(a) and Figure 2(b), where €(v)^H_v_^: a set of H_v_ hop distance neighborhood sensor nodes. Let ${\text{X}}_{€{\left(\text{v}\right)}^{{\text{H}}_{\text{v}}}}^{\text{T}}$ and ${\text{X}}_{€{\left(\text{v}\right)}^{{\text{H}}_{\text{v}}}}^{\text{G}}$ denote the total number of detectable €(v)^H_v_^ of N_v_ for Triangle-based and Grid-based respectively. According to Figure 2, we easily get:

(4) $${\text{X}}_{{€(\text{v})}^{{\text{H}}_{\text{v}}}}^{\text{T}}=3(1+{\text{H}}_{\text{v}}){\text{H}}_{\text{v}}$$

(5) $${\text{X}}_{€{\left(\text{v}\right)}^{{\text{H}}_{\text{v}}}}^{\text{G}}=2(1+{\text{H}}_{\text{v}}){\text{H}}_{\text{v}}$$

where H_v_ ≥1, we get ${\text{X}}_{€{\left(\text{v}\right)}^{{\text{H}}_{\text{v}}}}^{\text{T}}\gg {\text{X}}_{€{\left(\text{v}\right)}^{{\text{H}}_{\text{v}}}}^{\text{G}}$ that prove the Triangle-based approach is more suitable.

### 3.2. Assumptions

- Both SS and MS have buffers and queuing FIFO caches to store queuing data for *Round* communication.
- The time spent by MS fast movement can be technically ignored compared to the entire network time by using the proposed MS mobility model.
- Sensor failures are primarily caused by energy depletion.

### 3.3. Basic Definitions

**SS (Static Sink):** acts as a process center of the network.

**MS (Mobile Sink):** follows SS’s Responsibility Distribution (RD) assignment.

**Event Exploration (EE)** for *𝒯*_ɛɛ_ (time interval): Event Nodes (ENs) continuously send queuing data to SS at current event round j (ℛ_j_), when keeping idle for *𝒯*_ɛɛ_, SS treats the coming queuing data is from ℛ_j+1_ that indicates one single EE is declared to be finished.

**SS-Analysis (SSA)** for *𝒯*_SSA_: The data stored in SS buffer will be analyzed in a timelyway by SS, never being influenced by circumstance turbulence. Unexpected errors during SSA are beyond our consideration.

**Responsibility Distribution (RD)** for *𝒯*_RD_: After SSA, SS settle down its own responsibility by filtering the received data. While determining a certain amount of ENs within reachable Event Area (EA) to control based on residual energy and adjustable ratio frequency (to achieve different transmission distance), SS assign the rest of ENs to MS( ${𝒳}_{MS}^{\text{j}-1}$, ${𝒴}_{MS}^{\text{j}-1}$) with an optimal MS movement coordinate ( ${𝒳}_{MS}^{\text{j}}$, ${𝒴}_{MS}^{\text{j}}$). MS don’t need EE and SSA, just inherit SS’s assigned responsibility.

**MS-movement (MSmov)** for *𝒯*_MSmov_ : Once a new assignment was settled down, MS start calculating the destination with ( ${𝒳}_{MS}^{\text{j}}$, ${𝒴}_{MS}^{\text{j}}$) by analyzing the coordinates of its controlled ENs, and quickly moves from current ( ${𝒳}_{MS}^{\text{j}-1}$, ${𝒴}_{MS}^{\text{j}-1}$).

**MS-Analysis (MSA):** Once MS got RD assignment, it starts working on its own responsibility.

**Round System (RS)** for *𝒯*_ℛ_j__: A unit procedure including EE, SSA, RD, MSmov and MSA is called RS. The consumed time of ℛ_j_ is *𝒯*_ℛ_j__ in total. ℛ_j_ begins with a first queuing data from ENs arriving at SS and ends at the moment that all ENs are controlled either by SS or MS.

### 3.4. Energy Model in General

**Figure 3.**
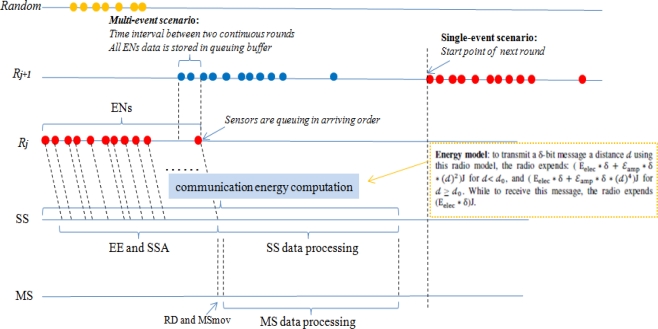
Processing map based on time coordinate.

Suppose a total of N sensors randomly distributed over a 2D terrain. To transmit a δ-bit message a distance *𝒹* using this radio model, the radio expends [21]: (E_elec_ * δ + ɛ_amp_ * δ *(*𝒹*)^2^)J for *𝒹*< d_0_, and ( E_elec_ * δ + ɛ_amp_ * δ *(*𝒹*)^4^)J for *𝒹* ≥ *𝒹*_0_.while to receive this message, the radio expends (E_elec_ * δ)J. The storage and query process is as similar as [5]. We give the whole processing map as described in Figure 3.

/*For simplicity, discussion is carried out at *𝒹* < d_0_ and Event Node (EN) denotes the active nodes.*/Time relationship:

(6) $${𝒯}_{{ℛ}_{\text{j}}}={𝒯}_{ɛɛ}+{𝒯}_{\text{SSA}}+{𝒯}_{\text{RD}}+{𝒯}_{\text{MSmov}}+{𝒯}_{\text{MSA}}$$

Energy relationship:

(7) $$E_{{𝒯}_{{ℛ}_{\text{j}}}}=E_{{𝒯}_{ɛɛ}}+E_{{𝒯}_{\text{SSA}}}+E_{{𝒯}_{\text{RD}}}+E_{{𝒯}_{\text{MSmov}}}+E_{{𝒯}_{\text{MSA}}}$$

(1) Energy consumption for EE:

(8) $$E_{{𝒯}_{ɛɛ}}=\sum_{𝒾=0}^{\text{N}}({\text{E}}_{\text{elec}}*\delta_{{\text{EN}}_{𝒾}^{\text{j}}}+{ɛ}_{\text{amp}}*\delta_{{\text{EN}}_{𝒾}^{\text{j}}}*{\left({𝒹}_{\text{SS}-{\text{EN}}_{𝒾}}^{\text{j}}\right)}^{2})+{\text{E}}_{\text{elec}}*\delta_{{\text{EN}}_{𝒾}^{\text{j}}}*\text{N}$$

where:

*𝒾* is the sensor index ( $𝒾\in \{1∼{\text{N}}_{\text{SS}}^{\text{j}}\}$)
j is the round index
$\delta_{{\text{EN}}_{𝒾}^{\text{j}}}$ shows corresponding size of the message that be used during the communication between ${\text{EN}}_{𝒾}^{\text{j}}$ and SS.
${𝒹}_{\text{SS}-{\text{EN}}_{𝒾}}^{\text{j}}$ is the distance between SS and ${\text{EN}}_{𝒾}^{\text{j}}$ in the current round.

(2) Energy consumption for SSA:

(9) $$E_{{𝒯}_{\text{SSA}}}=\sum_{𝒾=0}^{{\text{N}}_{\text{SS}}^{\text{j}}}({\text{N}}_{\text{SS}}^{\text{j}}*\bar{{𝒹}_{\text{SS}}^{\text{j}}})*{𝒞}_{\text{SS}}^{\text{j}}$$

where:

${\text{N}}_{\text{SS}}^{\text{j}}$ is the total number of ENs under SS’s control.
$\sum_{𝒾=0}^{{\text{N}}_{\text{SS}}^{\text{j}}}{\text{EN}}_{𝒾}^{\text{j}}$ is a set of ENs under SS’s controls.
$\bar{{𝒹}_{\text{SS}}^{\text{j}}}$ is the average distance between ${\text{N}}_{\text{SS}}^{\text{j}}$ and ${\text{EN}}_{𝒾}^{\text{j}}$.
${𝒞}_{\text{SS}}^{\text{j}}$ shows the capacity that indicates how much energy does SS cost to manage a unit distance(*𝒹*_unit_) data communication. The capability is determined in a priority. And the value of ${𝒞}_{\text{SS}}^{\text{j}}$ is proportional to its residual energy $E_{\text{SS}}^{\text{j}}$ and radio frequency ( ${\text{V}}_{\text{SS}-\text{fre}}^{\text{j}}$) at ℛ_j_:

(10) $${𝒞}_{\text{SS}}^{\text{j}}=f(E_{\text{SS}}^{\text{j}},\;{\text{V}}_{\text{SS}-\text{fre}}^{\text{j}})=\mu_{\text{SS}}^{E}{\text{E}}_{\text{SS}}^{\text{j}}+\mu_{\text{SS}}^{\text{V}}{\text{V}}_{\text{SS}-\text{fre}}^{\text{j}}+\eta_{\text{SS}}$$

where, $E_{\text{SS}}^{\text{j}}$ is the residual energy of SS at ℛ_j_.

$\mu_{\text{SS}}^{E}$, $\mu_{\text{SS}}^{\text{V}}$ *and* η_SS_ are the parameters.

(3) Energy consumption for RD.

(11) $$E_{{𝒯}_{\text{RD}}}=\sum_{𝒦=0}^{{\text{N}}_{\text{MS}}^{\text{j}}}({\text{E}}_{\text{elec}}*\delta_{{\text{EN}}_{𝒦}^{\text{j}}}+{ɛ}_{\text{amp}}*\delta_{{\text{EN}}_{𝒦}^{\text{j}}}*{\left({𝒹}_{\text{SS}-\text{MS}}^{\text{j}}\right)}^{2})+{\text{E}}_{\text{elec}}*\delta_{{\text{EN}}_{𝒦}^{\text{j}}}*\text{N}$$

where:

(12) $$\text{N}={\text{N}}_{\text{SS}}^{\text{j}}+{\text{N}}_{\text{MS}}^{\text{j}}$$

$𝒦\in \{1∼{\text{N}}_{\text{MS}}^{\text{j}}\}$
${\text{N}}_{\text{MS}}^{\text{j}}$ is the total number of ENs under MS’s control.
$\sum_{𝒦=0}^{{\text{N}}_{\text{MS}}^{\text{j}}}{\text{EN}}_{𝒦}^{\text{j}}$ is a set of ENs under MS’s control.

(4) Energy consumption for MSmov:

For the movement to the destination ( ${𝒳}_{MS}^{\text{j}}$, ${𝒴}_{MS}^{\text{j}}$) from current ( ${𝒳}_{MS}^{\text{j}-1}$, ${𝒴}_{MS}^{\text{j}-1}$). We have:

(13) $$E_{{𝒯}_{\text{MSmov}}}={𝒹}_{\left({𝒳}_{MS}^{\text{j}-1},\;{𝒴}_{MS}^{\text{j}-1})-({𝒳}_{MS}^{\text{j}},\;{𝒴}_{MS}^{\text{j}}\right)}*{𝒞}_{{𝒯}_{\text{MSmov}}}$$

${𝒞}_{{𝒯}_{\text{MSmov}}}$ shows the capacity that indicates how much energy does MS cost to manage a unit distance (*𝒹*_unit_) movement.

(5) Energy consumption for MSA:

(14) $$E_{{𝒯}_{\text{MSmov}}}=\sum_{𝒦=0}^{{\text{N}}_{\text{MS}}^{\text{j}}}({\text{N}}_{\text{MS}}^{\text{j}}*\bar{{𝒹}_{\text{MS}}^{\text{j}}})*{𝒞}_{\text{MS}}^{\text{j}}$$

where, ${𝒞}_{\text{MS}}^{\text{j}}$ shows the capacity that indicates how much energy does MS cost to manage a unit distance (*𝒹*_unit_) data analysis. The capability is determined in a priority. But the value of ${𝒞}_{\text{MS}}^{\text{j}}$ is proportional to its residual energy $E_{\text{MS}}^{\text{j}}$ at ℛ_j_, where, $E_{\text{MS}}^{\text{j}}$ is the residual energy of MS:

(15) $${𝒞}_{\text{MS}}^{\text{j}}=\mu_{\text{MS}}^{E}E_{\text{MS}}^{\text{j}}+\eta_{\text{MS}}$$

$\mu_{\text{MS}}^{E}$ and η_MS_ are the parameters.

(6) Key parameters for energy performance evaluation of SS and MS in Section 5:

(16) $${𝒹}_{\text{SS}-{\text{EN}}_{𝒾}^{\text{j}}}^{\text{j}}=\sqrt{{\left({𝒳}_{\text{ss}-{\text{EN}}_{𝒾}^{\text{j}}}^{\text{j}}-{𝒳}_{\text{ss}}^{\text{j}}\right)}^{2}+{{𝒴}_{\text{ss}-{\text{EN}}_{𝒾}^{\text{j}}}^{\text{j}}-{𝒴}_{\text{ss}}^{\text{j}})}^{2}}$$

(17) $$\bar{{𝒹}_{\text{SS}-{\text{EN}}_{𝒾}^{\text{j}}}^{\text{j}}}=\frac{\sum_{1}^{{\text{N}}_{\text{SS}}^{\text{j}}}{𝒹}_{\text{SS}-{\text{EN}}_{𝒾}^{\text{j}}}^{\text{j}}}{{\text{N}}_{𝒮𝒮}^{\text{j}}}$$

(18) $${𝒹}_{\text{MS}-{\text{EN}}_{𝒦}^{\text{j}}}^{\text{j}}=\sqrt{{\left({𝒳}_{\text{MS}-{\text{EN}}_{𝒦}^{\text{j}}}^{\text{j}}-{𝒳}_{\text{SS}}^{\text{j}}\right)}^{2}+{\left({𝒴}_{\text{MS}-{\text{EN}}_{𝒦}^{\text{j}}}^{\text{j}}-{𝒴}_{\text{SS}}^{\text{j}}\right)}^{2}}$$

(19) $$\bar{{𝒹}_{\text{MS}-{\text{EN}}_{𝒦}^{\text{j}}}^{𝓳}}=\frac{\sum_{1}^{{\text{N}}_{\text{MS}}^{\text{j}}}{𝒹}_{\text{MS}-{\text{EN}}_{𝒦}^{\text{j}}}^{\text{j}}}{{\text{N}}_{\text{MS}}^{\text{j}}}$$

## 4. Network Model

This section presents the network model in detail in a flexible way of representing objects and their relationships. Its distinguishing features are described in a graphical way.

### 4.1. Network Initialization

**Figure 4.**
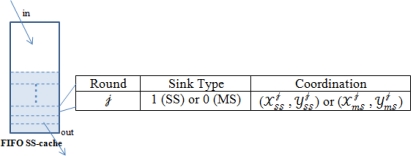
FIFO SS-cache.

SS is locaed at the center (50, 50) of the network area (100 m)^2^, while MS is at a random position on the network boundary waiting for RD. With the completion of sensors’ homogeneous distribution on the fixed network area, SS broadcasts a HELLO message (see Table 1) to all the sensors. Response messages (see Table 2) will arrive at SS and be put into FIFO SS-cache (see Figure 4). In this way, SS makes sense of all the active sensors at a high energy cost in the beginning. Then event happens continuously and connectively as described in Section 5. Technically we even have to face multi-event scenarios and event mergence problems that will be conducted in simulation part later.

### 4.2. MS Mobility Model (m = 1)

Intuitively, increasing the sink velocity *v* will improve the system efficiency, since in a unit time interval the mobile sink can contact more sensors and gather more information throughout the sensor field. We should carefully choose this parameter, explained as follows. On the one hand, the higher the MS velocity is, the higher the probability for sensors to meet MSs. On the other hand, when MSs are moving too fast across the effective communication region of sensors, there may not be a sufficiently long session interval for the sensor and sink to successfully exchange one potentially long packet. In other words, with the increase of MS velocity, the “outage probability” of packet transmission will arise. Therefore, finding a proper value for sink velocity must be a tradeoff between minimizing the sensor-MS meeting latency and minimizing the outage probability.

Suppose the network consists of *m* MSs and *n* sensors in a disk of unit size (of radius $1/\sqrt{\pi }$). Both the sink and sensor operate with transmission range of *r*. The mobility pattern of the mobile sinks M*_i_* (*i* = 1, ..., *m*) is according to “Random Direction Mobility Model” [22], however, with a constant velocity *v*. The sink’s trajectory is a sequence of epochs, and during each epoch the moving speed v of MS*_i_* is invariant and the moving direction of MS*_i_* over the disk is uniform and independent of its position.

Denote σ*_i_* as the epoch duration of MS*_i_*, which is measured as the time interval between MS*_i_*’s starting and finishing points. Q*_i_* is an exponentially distributed random variable, and the distributions of different σ*_i_* (i = 1, ...,*m*) are independent and identically-distributed (iid) random variables with common average of σ̄. Consequently the epoch length of different L*_i_*s are also iid random variables, sharing the same average of L = σ̄ × *v*.

**Figure 5.**
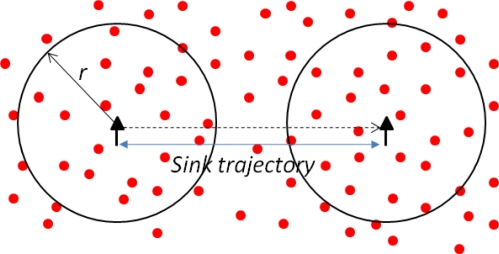
Computing the distribution of sensor-MS meeting delay.

Assume a stationary distribution of MSs, then the probabilities of independent MSs approaching a certain static sensor from different directions are equal. Specifically, the meeting of one static sensor N_j_ (j = 1, ..., n) and one mobile sink MS_i_ is defined as MS_i_ covers N_j_ during an epoch. Since MS_i_ will cover an area of size πr^2^+2r×L_i_, k (Figure 5) during the k-th epoch, then the number of epochs X_i_ needed till the first sensor-MS meeting is geometrically distributed with average of $\frac{1}{\text{p}}=\frac{1}{{\pi \text{r}}^{2}+2\text{r}+\bar{\text{L}}}$ (Theorem 3.1 of [22]), with the cumulative density function (cdf) as:

(20) $${\text{F}}_{{\text{X}}_{i}}(x)=\sum_{{\text{x}}_{\text{k}}\leq \text{x}}\text{p}{\left(1-\text{p}\right)}^{\text{k}-1}$$

where, p is a service probability.

In the case of multiple mobile sinks, the sensor-MS meeting delay should be calculated as the delay when the first sensor-MS meeting occurs. Thus the number of epochs X needed should be the minimum of all X*_i_* (i = 1, ..., *m*), with the cdf as:

(21) $${\text{F}}_{\text{x}}(\text{x})=1-{\left[1-{\text{F}}_{{\text{x}}_{\text{i}}}(\text{x})\right]}^{\text{m}}\approx \sum_{{\text{x}}_{\text{k}}\leq \text{x}}\text{mp}{\left(1-\text{p}\right)}^{\text{k}-1}$$

Denote X̄ as the average of X, then the expected sensor sink meeting delay will be:

(22) $${\text{D}}_{1}=\bar{\text{X}}\cdot \frac{\bar{\text{L}}}{v}$$

If we increase the radio transmission range *r*, or increase the number of MSs *m*, or increase the sink velocity *v*, the sensor sink meeting delay can be reduced. The above analysis has implicitly neglected the packet transmission delay during each sensor-MS encounters. However, if the message length is not negligible, the message has to be split into several segments and deliver to multiple sinks.

***Message delivery delay*** can be mainly attributed to the packet transmission time. In case of packet segmentations, the split packets are assumed to be sent to different sinks and reassembled. Assume the sensor will alternate between two states, active and sleep, whose durations will be exponentially distributed with a mean of 1/λ. Thus the message arrival is a Poisson process with arrival rate λ. For constant message length of L, constant channel bandwidth w, the number of time slots required to transmit a message is T = L·*w*. Then with a service probability p = mπr2, the service time of the message is a random variable with Pascal distribution (Lemma 1 of [7]). That is, the probability that the message can be transmitted within no more than x time slots, is:

(23) $${\text{F}}_{\text{x}}(\text{x})=\sum_{\text{i}=0}^{\text{x}-\text{T}}\left(\begin{array}{c} \text{T}+\text{i}-1\\ \text{T}-1 \end{array}\right){\text{p}}^{\text{T}}{\left(1-\text{p}\right)}^{\text{i}}$$

Such a Pascal distribution with mean value of $\frac{\text{T}}{\text{p}}=\frac{\text{L}}{{\pi \text{m}\omega \text{r}}^{2}}$. Under an average Poisson arrival rate λ and a Pascal service time with $\mu =\frac{\text{p}}{\text{T}}=\frac{{\pi \text{m}\omega \text{r}}^{2}}{\text{L}}$, data generation and transmission can be modeled as an M/G/1 queue. Then the average message delivery delay can be expressed as follows:

(24) $${\text{D}}_{2}=\frac{1}{\lambda }[\rho +\frac{\rho^{2}+\lambda^{2}\rho^{2}}{2(1-\rho )}]$$

where $\rho =\frac{\lambda }{\mu }$, for simplicity, we neglect the impact of arrival rate and set λ = 1, thus:

(25) $${\text{D}}_{2}=\frac{1}{\mu -1}=\frac{1}{\frac{\pi \text{m}\omega {\text{r}}^{2}}{\text{L}}-1}$$

This result shows that, by decreasing message length L, or increasing transmission range *r* and number of mobile sinks m, the message delivery delay can be reduced. However, for simplicity the value of m was assigned to be “1” for the forthcoming discussions.

### 4.3. Single Event Scenario

Assume that only one event happened at any moment. The ENs send queuing messages (see Table 2 & 3) to SS one by one (see Figure 1). Finally, SS get all the information of ( ${\text{N}}_{\text{SS}}^{\text{j}}+{\text{N}}_{\text{MS}}^{\text{j}}$). In order to save energy and take a full advantage of MS’s existence, the network is pursuing a perfect performance combination of SS and MS. As a result, we set a threshold distance ${𝒹}_{\text{SS}-\text{threshod}}^{\text{j}}$ for SS based on its residual energy $E_{\text{SS}}^{\text{j}}$:

(26) $${𝒹}_{\text{SS}-\text{threshod}}^{\text{j}}=f(E_{\text{SS}-\text{fre}}^{\text{j}})$$

#### a. SS inside the event

**Figure 6.**
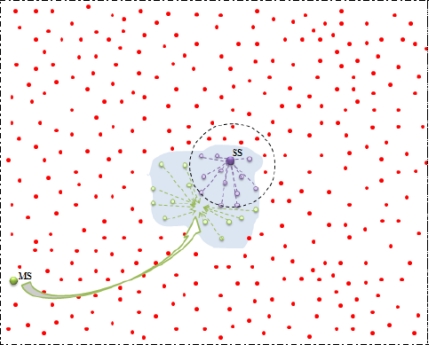
SS inside the event (MSmov).

After SS ( $E_{\text{SS}-\text{fre}}^{\text{j}}$) fixed, SS get knowledge about all the ENs under their own control (see Figure 6). Therefore SS start working on its responsibility and performs RD.

After RD assignment, MS move to the destination at a certain velocity *v* and then give a feedback (ACK) to SS (see Table 4) with its current coordinate information (see Table 5), meanwhile multicast to all the assigned sensors in green to let them know its position and status (see Table 6).

#### b. SS outside the event

**Figure 7.**
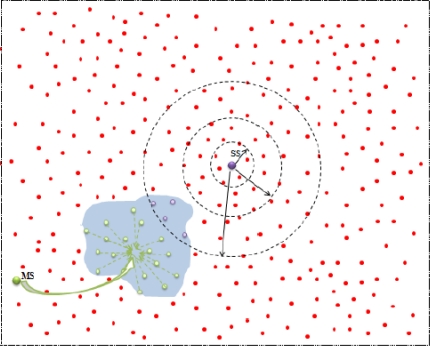
SS outside the event (MSmov).

Figure 7 shows that SS is controlling the ENs in violet, while MS is controlling the ENs in green. Once RD is distributed, both of SS and MS are working on their own ENs.

### 4.4. Multi-Event Scenario

**Figure 8.**
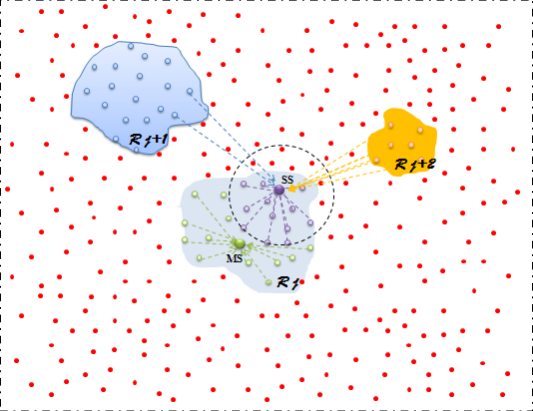
Multi-event scenario.

Assume that multi-events happen synchronously. For simplicity, we explain the phenomena of 3-event co-existence (see Figures 3 and 8). The sensors in green are ℛ_j_ ENs. Some sensors are those whose HELLO message arrive at both SS and MS during ℛ_j_. In this case, MS ignore these messages but SS will regularly work on them. A more complex situation is that ENs in blue (ℛ_j+1_) and in yellow (ℛ_j+2_), all the ℛ_j_ ENs arrive at SS. In this case, after performing EE and SSA, SS will temporarily let sensors at ℛ_j+1_ and ℛ_j+2_ to wait until MS is free (ℛ_j_ is finished). Once MS finished its assigned RD, it reports to SS. If there are multi-event happening at the same time, EE and SSA is as normal. However, the RD assignment to MS is different from that of single event scenario. We make event-based RD assignment that MS randomly deals with events one by one.

## 5. Performance Evaluation

We evaluate the performance of the eDSA implemented in the C++ simulator. Each sensor has limited resources and is equipped with an Omni-directional antenna.

**Figure 9.**
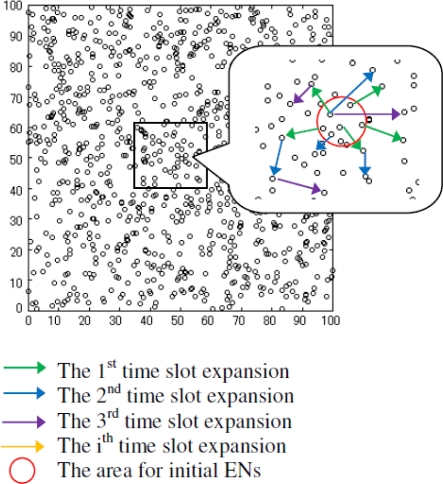
Single-event model with the initial ENs that adequately covers a circle area {(x−10)^2^+(y−10)^2^=R_circle_^2^} to generate the event.

- Single-event model (see Figure 9): the initial ENs that adequately covers a randomly selected circle area {(x−10)^2^+(y−10)^2^=R_circle_^2^} to irritate the event. At every time slot, EN propagates by picking up a random number of neighbors to join the event (non-ENs→ENs). In this way, the event is guaranteed to be fully connected.
- Multi-event model: randomly picks up three points that should be geo-separated over at least 50 m distance in the coordinate and then initiate events in a similar way as in the single-event model.

### Energy consumption:

**Figure 10.**
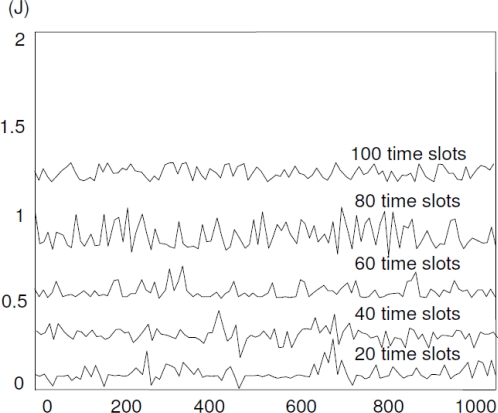
Energy consumption status of 1,000 sensors randomly distributed over a 100*100 square meters network area under single-event scenario, associated with Figure 11.

In Figure 10, we captured the energy level status of all the 1,000 sensors under a single-event scenario at 20, 40, 60, 80,100 time slots, respectively. The results represent the average performance of our proposed network over 100 simulation trials. Obviously, it differs every time, but this makes no difference. For the first 20 time slots, the overall energy level curve is flat, however we still find something special that sensors with index around 610∼660 cost more energy than others. This might be caused by the occurrence of the single event. Moreover, it is shown that the average energy consumption increased at every 20 time slots which is due to the expansion of the event area that make more and more sensors involved and become ENs.

The biggest difficulty in object tracking in WSNs is to manage multi-event scenario where events merge into a lesser number of events to form a more complicated network phenomena. We focused on the energy performance of the SS and MS which are more critical than other ordinary sensors for the whole monitoring network.

**Figure 11.**
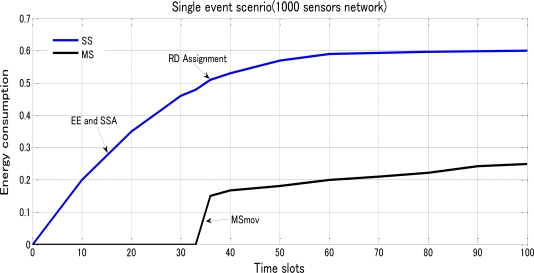
Energy consumption of SS and MS based on the spent time lots under a single-event scenario with totally 1,000 sensors randomly distributed over a 100*100 network area.

**Figure 12.**
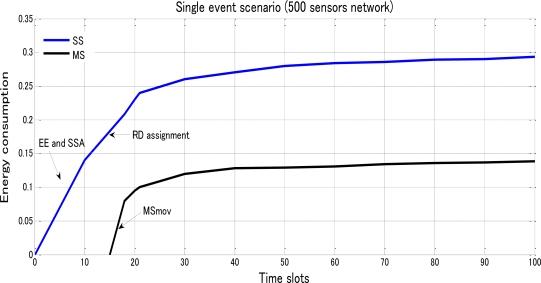
Energy consumption of SS and MS based on the spent time slots under a single-event scenario with 500 sensors randomly distributed over a 100*100 network area.

In Figure 11, we got a curve in blue for SS to show exponential increase with a sudden weak-downturn at RD assignment and a curve in black for MS to show a calm attitude towards the first approximate 33 time slots, a sudden ascending caused by MSmov and more smooth exponential increase in energy consumption. Moreover we varied the number of sensors with 500 in total, the energy performance was reduced by nearly half proven by Figure 12.

**Figure 13.**
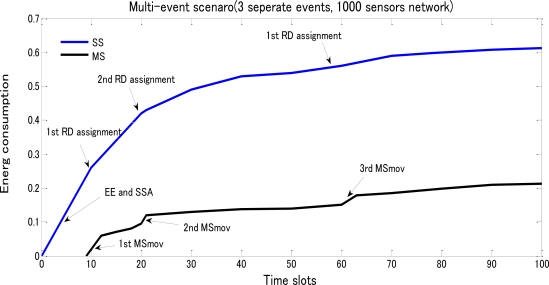
Energy consumption of SS and MS based on the spent time lots under a 3-event scenario with totally 1,000 sensors randomly distributed over a 100*100 network area.

Figure 13 gives the energy performance of SS and MS based on a 1,000 sensors network with totally three events randomly generated according to the multi-event model. As expected, the energy performance for MS has three sudden ascending steps at three different timings (9, 21, 60 time slots respectively). However, for SS, the overall performance keeps similar due to its requirements on movements.

## 6. Conclusions

In this paper, we have explored an optimal barrier coverage based sensor deployment for object tracking WSNs where a dual-sink model was designed to evaluate the energy performance of all the ordinary sensors and Static Sinks (SSs) and Mobile Sinks (MSs) simultaneously, based on parameters such as sensor transmission range *r* and the velocity of the mobile sink *v*. Moreover, we purposely designed a mobility model for MSs for the proposed dual-sink eDSA algorithm using the knowledge of the cumulative density function (cdf), Poisson process and M/G/1 queue. The simulation results show the energy performance for the whole network clearly and verify that the eDSA is more efficient by reducing approximately by half the energy consumption compared with a one static-sink model. Our future work will include verification of the precision of MS trajectories [23] and invention of a new protocol that considers the fast mobility of each sensor as well as destructive sensors or sudden failures in the network connectivity during communication.

## Figures and Tables

**Table 1.** Hello message (for both SS and MS).

**Round**	**Sink Type**	**Coordination**
j	1 (SS) or 0 (MS)	( ${𝒳}_{\text{SS}}^{\text{j}}$, ${𝒴}_{\text{SS}}^{\text{j}}$) or ( ${𝒳}_{MS}^{\text{j}}$, ${𝒴}_{MS}^{\text{j}}$)

**Table 2.** Response (queuing) message.

**Sink Type**	**Coordinate**
1 (SS) or 0 (MS)	( ${𝒳}_{\text{SS}-{\text{EN}}_{𝒾}^{\text{j}}}^{\text{j}}$, ${𝒴}_{\text{SS}-{\text{EN}}_{𝒾}^{\text{j}}}^{\text{j}}$) or ( ${𝒳}_{\text{MS}-{\text{EN}}_{𝒦}^{\text{j}}}^{\text{j}}$, ${𝒴}_{\text{MS}-{\text{EN}}_{𝒦}^{\text{j}}}^{\text{j}}$)

**Table 3.** SS self-RD assignment.

1	Input: Input: G = (V, E) while E≠∅ do
2	Loop Function EE and SSA
3	until SS got all the ENs
4	E_*𝒯*_ɛɛ_j___ (8) and E_*𝒯*_SSA__ (9)
5	If ${𝒞}_{\text{SS}}^{\text{j}}=\text{TRUE}\;\text{then}\;\to \text{SS}\;({\text{V}}_{\text{SS}-\text{fre}}^{\text{j}})$
6	Else if ${𝒞}_{\text{SS}}^{\text{j}}=\text{FALSE}\;\text{then}\;\text{terminate}$
7	Else go to loop
8	End if
9	End

**Table 4.** SS→MS.

**Sink Type**	**Content**
1(SS)	RD

**Table 5.** Feedback (ACK) MS→SS.

**Sink Type**	**Current coordinate**
0(MS)	${𝒳}_{MS}^{\text{j}}$, ${𝒴}_{MS}^{\text{j}}$

**Table 6.** MS→ ${\text{EN}}_{\text{𝒦}}^{\text{j}}$.

**Sink Type**	**Content**
0(MS)	1

**Table 7.** Simulation parameters.

***Parameter***	**Value**

Network Area	100 m^2^
The location of SS	(50,175),
Transmission range (r)	20 m
Time slots(T)	100 (seconds)
Initial Energy/sensor	2J/battery
All the coefficients ( $\mu_{\text{SS}}^{E}$, $\mu_{\text{SS}}^{\text{V}}$, η_SS_, $\mu_{\text{MS}}^{E}$ and η_MS_*etc.*)	1
Message size (L)	100 Bytes
MS Velocity(*v*) with *m*=1	5∼10 m/sec
E_elec_	50 nJ/bit
E_fs_	10 pJ/bit/m2
ɛ_amp_	0.0013 pJ/bit/m4
E_DA_	5 nJ/bit/signal
***𝒹***_**0**_	80 m

## References

[1] Cardei M, Wu J (2004). Coverage in wireless sensor networks. Handbook of Sensor Networks: Compact Wireless and Wired Sensing Systems.

[2] Meguerdichian S, Koushanfar F, Potkonjak M, Srivastava MB Worst and best-case coverage in sensor networks.

[3] Li XY, Wan PJ, Frieder O (2003). Coverage in wireless ad-hoc sensor networks. IEEE Trans Comput.

[4] Chen JH, Kim CS, Song F A Distributed Clustering Algorithm for Voronoi Cell-based Large Scale Wireless Sensor Network.

[5] Heinzelman W, Chandrakashan A, Balakrishnan H (2002). An application-specific protocol architecture for wireless microsensor networks.

[6] Huang H, Richa AW, Segal M (2005). Dynamic coverage in ad-hoc sensor networks. Mobile Networks and Applications.

[7] Gaotao S, Liao MH Exploiting sink movement for energy-efficient load-balancing in wireless sensor networks.

[8] Luo J, Hubaux JP Joint mobility and routing for lifetime elongation in wireless sensor networks.

[9] Xie DL, Chen MX, Chen CF, Ma J A Patrol Grid Protocol for Mobile Wireless Sensor Network.

[10] Ye F, Luo HY, Cheng J, Lu SW, Zhang LX (2002). A two-tier data disseminication model for large scale wireless sensor networks.

[11] Cheng L, Gao JN, Chen CF, Chen HY, Ma J, Joanna IS Cooperative Contention-Based Forwarding for Wireless Sensor Networks.

[12] Wang B, Xie DL, Chen CF, Ma J, Cheng SD Deploying Multiple Mobile Sinks in Event-Driven WSNs.

[13] Chen CF, Ma J MEMOSEN: Multi-radio Enabled MObile Wireless SEnsor Network.

[14] Cheng L, Chen YM, Chen CF, Ma J Query-Based Data Collection in Wireless Sensor Networks with Mobile Sinks.

[15] Biao R, Ma J, Chen CF The Hybrid Mobile Wireless Sensor Networks for Data Gathering.

[16] Chen CF, Ma J, Salomaa J Simulation study of cluster based data dissemination for wireless sensor networks with mobile sinks.

[17] Wang Y, Wu HY (2006). DFT-MSN: The Delay/Fault-Tolerant Mobile Sensor Network for Pervasive Information Gathering.

[18] Wang B, Xie DL, Chen CF, Ma J, Shiduan C Employing Mobile Sink in Event-Driven Wireless Sensor Networks.

[19] Wang GJ, Wang T, Jia WJ, Guo MY, Li J (2009). Adaptive location updates for mobile sinks in wireless sensor networks. J Supercomput.

[20] Wang W, Srinivasan V, Chua K-C (2005). Using mobile relays to prolong the lifetime of wireless sensor networks.

[21] Heinzelman WR, Chandrakasan A, Balakrishnan H Energy-Efficient Communication Protocol for Wireless Microsensor Networks.

[22] Thrasyvoulos S, Konstantinos P, Cauligi R Performance analysis of mobility-assisted routing.

[23] Chen HY, Shi QJ, Tan R, Poor HV, Sezaki K Mobile element assisted cooperative localization for wireless sensor networks with obstacles. IEEE Trans Wireless Comm.

